# Sleeping Less Than Usual Is Associated With Greater Daily Depression and Higher Reactivity to Negative Interpersonal Events Among Suicidal Adolescents

**DOI:** 10.1016/j.jadohealth.2026.02.009

**Published:** 2026-03-14

**Authors:** Delainey L. Wescott, Tina R. Goldstein, Joud Hijazi, Dawn Rice, Noelle Rode, Kim Poling, Jamie Zelazny, David Brent, Peter L. Franzen

**Affiliations:** a Department of Psychiatry, University of Pittsburgh School of Medicine, Pittsburgh, Pennsylvania; b Department of Nursing, University of Pittsburgh, Pittsburgh, Pennsylvania

**Keywords:** Sleep, Depression, Adolescence, Affective functioning

## Abstract

**Purpose:**

Changes in sleep can impact cognitive and emotional functioning and may contribute to depression. The current longitudinal study included a high-risk sample of adolescents and young adults with depression and/or suicidal thoughts and behaviors who were undergoing intensive outpatient treatment programs. We examined changes in sleep throughout treatment and whether daily changes in sleep impacted next-day depression through altered affective reactivity to interpersonal situations.

**Methods:**

Participants (N = 198, ages 13–23, 82% White, 76% female sex at birth) were recruited from intensive treatment programs for depression and suicide risk. Participants wore an actigraph and completed daily ratings of depression, sleep, and positive and negative reactivity to interpersonal events throughout treatment (M = 60 days, SD = 22 days). Clinicians rated weekly depression severity. Multilevel models tested changes in sleep and depression throughout treatment and examined daily relationships between total sleep time (TST), next-day depression, and affective reactivity. Multilevel mediation tested whether increased affective reactivity mediated daily relationships between TST and depression.

**Results:**

Throughout treatment, self-reported TST increased (b = 0.01; *p* < .001) and daily self-reported depression severity decreased (b = −0.06; *p* = .02), although actigraphy-assessed TST did not change (b = 0.01; *p* = .16). Sleeping less than usual on diaries (b = −0.29; *p* = .02) but not actigraphy when controlling for covariates (b = −0.19; *p* = .18) was related to worse next-day depression. Greater clinician-rated depression severity amplified the effects of short TST on next-day depression for both diary (b = −0.22; *p* = .03) and actigraphy-assessed TST (b = −0.32; *p* = .01). Increased negative affectivity statistically mediated daily associations between short TST and increased next-day depression (indirect effects: B = 0.03; *p* < .001).

**Discussion:**

Short sleep may contribute to depression through increased negative affectivity to interpersonal events. Future work should test whether sleep interventions in high-risk samples hastens symptom improvement.

Rates of depression increase during adolescence [1], and predict risk of subsequent depression into adulthood [2]. Adolescent depression is highly recurrent, with approximately half experiencing episode recurrence within 5 years [3]. Understanding factors that maintain depression symptomology may aid intervention efforts. Sleep disturbances, including short sleep duration, are common symptoms of adolescent depression [4] that often precede depression onset [5]. Further, sleep disturbances are residual symptoms following episode remission that predict future episode recurrence [6], suggesting disrupted sleep may both initiate and prolong depression symptomatology. The current study examined longitudinal changes in sleep and depression in a sample of adolescents and young adults at high risk for suicide receiving intensive outpatient treatment for depression and suicide risk, as well as prospective relationships between nightly sleep and next-day depression.

Adolescents are chronically sleep restricted [7] and nationally representative data from the United States suggest sleep duration in teens has been steadily declining [8]. Meta-analytic findings link insufficient sleep with decreased positive affect (PA), increased negative affect (NA), and depressed mood in typically developing adolescents [9]. Although related, affect captures transient emotional states in a particularly moment, and depressed mood encompasses cognitive, emotional, and reward-related states across time [10]. In community samples, adolescents with internalizing symptoms showed a stronger relationship between self-reported nightly sleep duration and next-day mood symptoms, suggesting sleep may be more impactful for daily depressed mood in symptomatic youth [11]. However, prior work in clinical samples have largely focused on adults and measured PA or NA, instead of depressed mood. Sleeping less than one’s usual amount has been associated with increased next-day NA, which was amplified in adults with moderate-to-severe depression [12]. Similarly, retrospective sleep disturbances and poor sleep efficiency have been associated with greater next-day NA to unpleasant events in adults with and without clinical depression. However, adults with depression reported elevated NA regardless of event valence, suggesting elevated, nonspecific NA in depression following sleep loss [13]. We aim to extend prior work by examining daily relationships between sleep and depressed mood in adolescent and young adult clinical samples. Based on prior work in adults, [12] depression severity may modulate daily sleep and mood relationships. Individuals with moderate-to-severe depression may be more susceptible to nightly changes in sleep duration―a possible pathway by which disrupted sleep prolongs depression. Insufficient sleep may contribute to, and exacerbate, depression through compromised affective functioning, an overarching term comprising mood states, emotions, and emotion regulation [10]. Experimentally induced sleep loss adversely impacts emotional arousal and emotion regulation, particularly in youth and adolescents [14]. Heightened negative emotions coupled with decreased capacity to regulate emotions may be a pathway by which insufficient sleep maintains depression. For example, short sleep during adolescence predicts depression onset in young adulthood, potentially through increased NA [15]. Altered affective functioning following insufficient sleep may be particularly potent for adolescents in social contexts, given increased sensitivity to social evaluation during this developmental period [16]. Pupillary affective reactivity, indexed by changes in pupil size, is one way to assess emotional reactivity, and is particularly sensitive to sleep loss [17]. Experimentally sleep-restricted adolescents demonstrated increased pupillary affective reactivity and negative affective behavior in social contexts [18], suggesting affective functioning in social contexts may be particularly salient during adolescence relative to both nonsocial contexts and adulthood [16]. In a prior subsample of the current analysis, we found that heighted affective reactivity to negative interpersonal events indirectly mediated daily associations between short sleep and suicidal ideation [19]. These findings controlled for prior-day depression to isolate the sleep―suicide association; daily variations in sleep may impact suicide risk and depression through shared and unique pathways. We aimed to extend these findings examining daily relationships between sleep, depression, and affective functioning in a larger high-risk clinical sample.

Sleep is largely under-targeted in high acuity clinical care. In outpatient settings, evidence-based psychotherapies, such as cognitive behavioral therapy and dialectical behavior therapy, have been associated with improved sleep during treatment [20–22]. These clinical improvements in sleep disturbances are largely limited to subjective sleep reports and diminish following treatment. It is unclear whether actigraphy-assessed sleep changes during intensive outpatient treatment given prior work has been limited to outpatient settings and by self-reports of sleep. To address this gap, the current study examined changes in self-reported and actigraphy-assessed sleep duration in adolescents and young adults receiving intensive outpatient treatment focused on depression and suicide risk.

The current prospective study examined daily changes in sleep and depression longitudinally in a sample of adolescents and young adults receiving treatment from an intensive outpatient program (IOP) or adolescent acute partial hospitalization program (AAPHP) for depression and suicide risk. We tested if prospective self-report and behavioral measures of sleep and self-report and clinician-rated depression changed across treatment. We hypothesized depression would improve and sleep duration would lengthen over the course of treatment. We further tested if daily changes in sleep behaviors related to next-day depression and whether depression severity moderated this relationship. We hypothesized that sleeping shorter than usual would be associated with greater next-day depression, particularly for participants with greater clinician-rated depression.

## Methods

### Participants

Adolescents and young adults receiving treatment in an IOP or AAPHP for depression and suicidality were recruited for the current study from 2018 to 2019 (study 1: ages 13–24) and from 2021 to 2024 (study 2: ages 13–18). IOP consisted of 9 hours of weekly treatment (3 hours/day, 3 days/week) and the AAPHP involved 24 hours of weekly treatment (6 hours/day, 4 days/week); both clinics involved skills groups, individual psychotherapy, and medication management. Clinical staff referred participants to the current study during their first month of IOP or AAPHP treatment. Participation in the study was voluntary and separate from their intensive clinical care services. Participants were eligible if they had access to a cellphone or computer at home. Participants provided informed consent and if under age 18 provided assent and had a parent/caregiver consent. Study was approved by the University of Pittsburgh IRB (study 1: approved January 19, 2018: #17070490; study 2: approved February 2, 2021: #20110313).

### Procedures

Participants completed clinical interviews and self-report questionnaires at baseline and monthly follow-up assessments throughout the duration of their treatment period conducted by master’s level evaluators trained to reliability on study measures. During the baseline visit, participants were instructed on how to complete daily surveys and provided with an actigraph. Participants were asked to complete daily surveys and wear the actigraph for up to 3 months (study 1), 2 months (study 2), or until discharge from care within the clinic. Participants were compensated for completing daily surveys and wearing the actigraph. Due to varying lengths of treatment, the number of available days differed across participants.

### Measures

#### Clinical assessments.

Electronic medical records provided initial diagnostic information from participants’ clinical intake assessment. After enrolling in the current study, clinical diagnoses/presentations were confirmed using the clinician-administered Adolescent Longitudinal Follow-up Evaluation Psychiatric Severity Rating scales [23]. Participants completed clinical research interviews monthly while enrolled in treatment, which allowed for weekly diagnostic depression ratings for the prior month. A primary outcome in this report was the Psychiatric Severity Rating-depression scale, rated on a 1–6 scale: scores of 1–2 indicate minimal depression (mild); 3–4 subthreshold depression (marked); and 5–6 met full diagnostic criteria for major depression (severe).

#### Behavioral sleep measurement.

Wrist-worn actigraphy was used to collect daily behavioral sleep data. Participants were asked to continuously wear an actigraph on their nondominant wrist (except when swimming): study 1: GT9x Link actigraphs (Artemis, formerly ActiGraph Corp, Pensacola, FL), study 2: CentrePoint Insight Device (Artemis, Pensacola, FL). Data were collected in 60-second epochs and scored using the Cole-Kripke algorithm [24,25] in the ActiLife software. The algorithm has been validated against polysomnography and other actigraph devices in both adolescents and adults [25]. ActiLife sleep detection settings included a bedtime definition of 10 minutes and wake time definition of 20 minutes in study 1 and 10 minutes in study 2. We visually inspected all actigraphy data for periods of nonwear time, verification of naps, and inconsistencies between the algorithm set major sleep periods and activity levels, including nonwear time within the algorithm detected sleep period, low-level activity for >10 minutes at the start or end of the major sleep period, or high levels of activity between nonwear periods detected as a nap. Sleep diaries were occasionally reviewed to aid the scoring algorithm but were not prioritized in the actigraphy cleaning process. In study 1, actigraphs were charged every 1–2 weeks during in-person clinical visits. In study 2, actigraphs were charged monthly at participants’ homes. Sleep onset time and total sleep time (time spent asleep during primary sleep episodes) were the primary sleep variables.

#### Daily surveys.

Participants completed daily morning and evening surveys through a secure web-based platform via links sent by SMS text message or email. Participants were trained on how to complete the daily surveys by research assistants, including examples of daily interpersonal events, and how to use the subjective ratings scales. Morning surveys were scheduled 30 minutes after habitual weekday and weekend wake times and included a subjective assessment of the prior night’s sleep [26]. Sleep diary questions included the time they first got into bed, when they tried to sleep “lights out,” sleep onset latency, the time they woke up, and number and duration of night-time awakenings. Total sleep time was calculated as the total time spent asleep between “lights out” and wake time.

Evening surveys were sent 45 minutes before habitual weekday and weekend bedtime and included a daily depression severity rating (“The worst my depression was today”) on a Visual Analog Scale ranging from 1 (“I didn’t feel depressed at all”) to 100 (“Horrible, the worst it gets”). Participants also rated their reactivity to both negative events (“How stressful/upsetting was your most negative event (conflict/excluded/insulted) today?”, rated on a scale of 1 “Not at all upsetting” to 100 “Extremely upsetting”) and positive events (“How enjoyable/pleasant was your most positive event (had fun/was complimented) today?”, rated on a scale of 1 “Not at all enjoyable” to 100 “Extremely enjoyable.” Negative (conflict/excluded/insulted) and positive (had fun/was complimented) events were derived the interpersonal domain of events of the Adolescent Life Events Questionnaire [27]. Participants reported whether their events included family, peers, other adults, or not applicable. Detailed description of the affective reactivity item construction has been published previously [19]. See Figure S1 for histograms of the Visual Analog Scale items from the combined study 1 and study 2 samples.

### Analytic plan

All analyses were conducted in R Studio (Version 2024.12.0 + 467). Multilevel models were used to account for repeated daily assessments within participants as indicated by significant within-person variability in primary outcomes (Intraclass Correlation Coefficients: 0.21–0.38). Models were fitted using the *nlme* package using maximum likelihood. We included a random intercept of participant and a random slope of time (e.g., day in study) to account for individual variation in primary study variables and in the rate of change across time. Likelihood ratio tests supported the inclusion of random slopes. We visually inspected primary outcomes to confirm linear changes over time. We person-centered continuous sleep predictors to calculate within-person nightly fluctuations in sleep. Between-person sleep averages were included as covariates. Level 1 covariates (time varying, within-person) included day in study, prior-day depression, and a weekday/weekend binary variable. Level 2 covariates (person varying, between-person) included age, sex at birth, and study protocol (study 1 or study 2). We also tested whether weekly clinician-rated depression severity moderated daily relationships between sleep and depression. We ran a sensitivity analysis removing any affective responses that did not include family, peers, or other adults and a separate sensitivity analysis removing young adults (N = 19). Repeated measures correlation using the *rmcorr* [28] package were used to test associations between daily survey variables: daily depression and positive/negative affectivity.

We tested indirect effects of within-person sleep fluctuations on next-day depression through interpersonal affectivity using 1-1-1 multilevel mediation in the *lavaan* package. Direct effects (*c’ path*) of within-person sleep fluctuations and next-day depression were calculated as main effects. We restricted mediation analyses to significant relationships between within-person sleep and interpersonal affectivity (*a’ paths*). Within-person fluctuations in sleep were included as a level 1 predictor, interpersonal affectivity measures as level 1 mediators, and next-day depression as level 1 outcome. Level 1 covariates included day in study, prior-day depression, and a weekday/weekend binary variable. Level 2 covariates included between-person averages of sleep and interpersonal affectivity, age, sex at birth, and study protocol. We ran separate mediation models for diary and actigraphy assessed sleep duration. Study results did not change after implementing the Benjamini–Hochberg procedure to correct for multiple comparisons [29].

## Results

### Sample

The current study included 198 participants aged 13–23 (82% White, 76% female sex at birth) from study 1 (N = 59) and study 2 (N = 139). See Table 1 for demographic information. There were 9,904 total available days for analysis (M = 60.2, Standard deviation = 22.2). Number of days in the study was not associated with clinician-rated depression (r = −0.04; *p* = .638). Participants completed 78.1% of expected morning surveys (M = 59.9 days, SD = 22.1, 9–121 days) and 78.0% of evening surveys (M = 60.0 days, SD = 22.2, 9–121 days). Daily survey data were missing for 2,159 mornings (21.8%), 2,179 evenings (22.0%), and were not available for 295 mornings (2.9%) and 351 evenings (3.5%) due to expected participant factors (e.g., hospitalization or technological problems with the surveys). Morning survey data were removed from sleep diary analyses for data quality issues (n = 39, <1%) and for missing sleep data (n = 134, 1.3%). Actigraphy data were available for 82.0% of expected days (M = 59.5, SD = 21.9, 9–121 days). Actigraphy data were missing for 1,682 days (16.9%), were not available for 494 days (4.9%) due to expected participant factors, and for 35 days (<1%) due to equipment failure.

### Changes in sleep and depression across treatment

**Figure 1.**
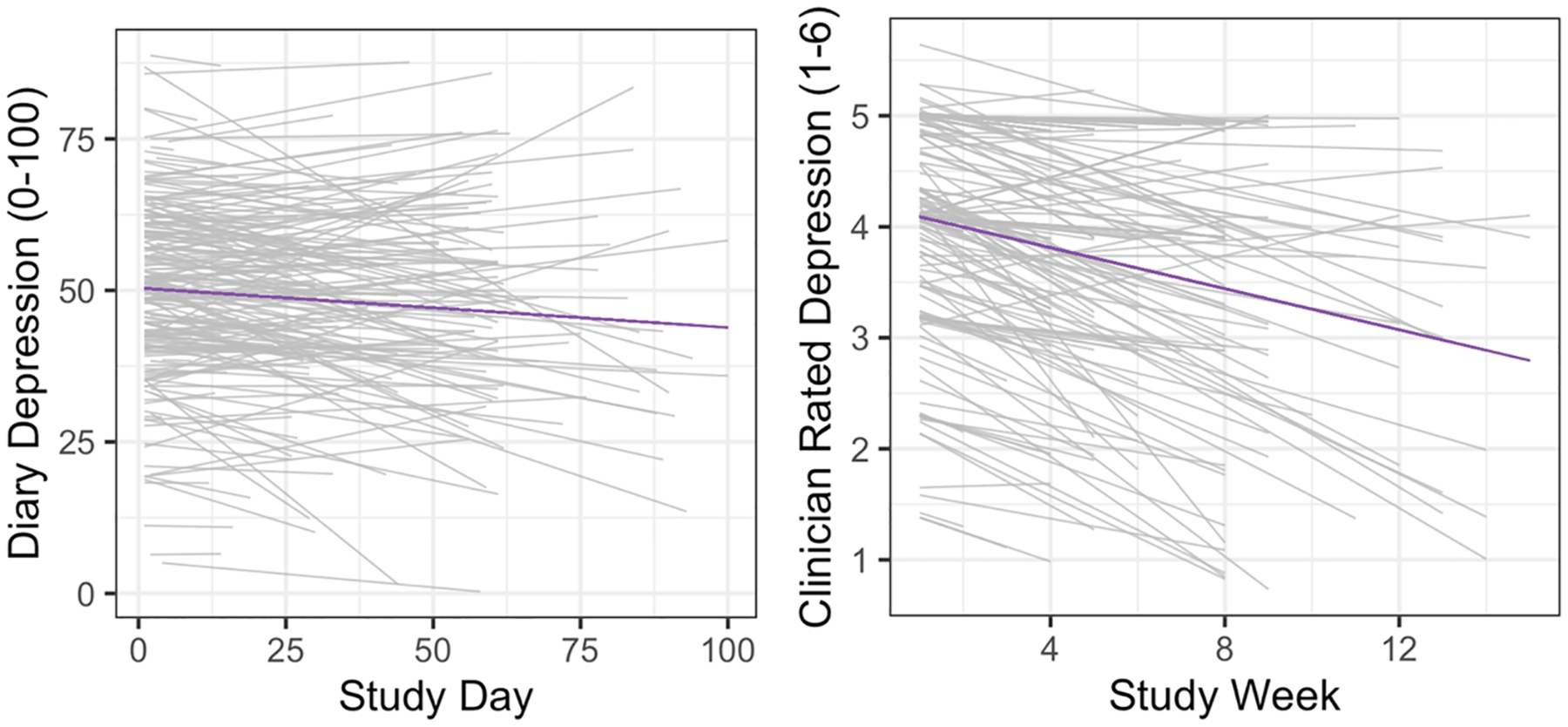
Changes in daily self-reported depression and weekly clinician-rated depression over time in intensive treatment. Gray lines represent slopes for individual participants. Purple lines represent overall average.

Daily self-reports (b = −0.06; *p* = .02) and weekly clinician ratings (b = −0.09; *p* < .001) of depression improved throughout treatment (Table S1, Figure 1). Self-reported total sleep time increased throughout treatment (b = 0.01; *p* < .001) while actigraphy-assessed total sleep time did not change (b = 0.00; *p* = .56; Table S2). Sleep onset timing did not change over the course of treatment (Table S3) while wake times became later over time on both diaries (b = 0.01; *p* = .02) and actigraphy (b = 0.01; *p* = .007; Table S4).

### Daily relationships between sleep, affective reactivity, and depression

**Figure 2.**
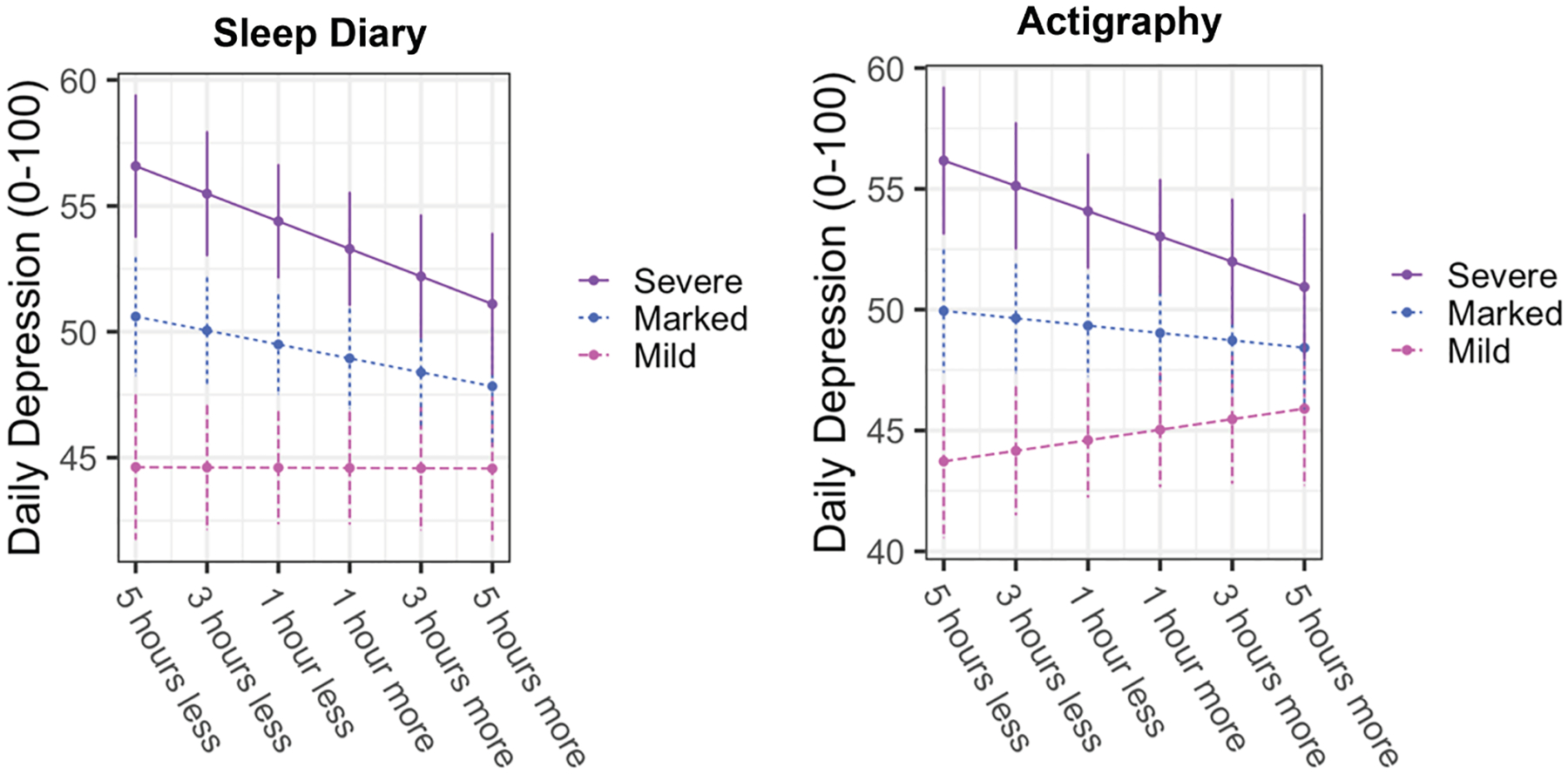
Weekly clinician-rated depression moderates the relationship between nightly changes in total sleep time and next-day depression for both sleep diaries (left) and actigraphy (right). Associations between within-person total sleep time and next day depression only significant at marked to severe clinician-rated depression (PSR 4–6) using simple slope analyses. PSR = Psychiatric Severity Rating scale.

Daily depression was moderately correlated with negative (r_rm_ = 0.52; *p* < .001) and positive affectivity (r_rm_ = −0.31; *p* < .001). Daily changes in total sleep time were associated with next-day self-reported depression. Sleeping less than a person’s usual on sleep diaries (b = −0.35; *p* = .01) was associated with greater next-day depression controlling for prior-day depression (Table 2). This finding did not hold for actigraphic total sleep time when including age, sex at birth, and study as covariates (b = −0.23; *p* = .14). The relationship between total sleep time and next-day depression severity was moderated by weekly clinician-rated depression (sleep diaries: b = −0.23; *p* = .03 | actigraphy: b = −0.31; *p* = .01). Simple slopes analyses indicated that individuals with marked-to-severe clinician-rated depression from that week showed a stronger negative relationship between total sleep time and depression such that sleeping less than usual was associated with worse next day depression (Figure 2). Daily changes in sleep onset timing were not associated with next day depression (Table S5). Sleeping less than usual on both diaries (b = −0.80; *p* < .001) and actigraphy (b = −0.84; *p* < .001) was associated with greater next-day negative interpersonal affectivity (Table 2). Daily changes in total sleep time were not associated with positive interpersonal affectivity (Table S6).

### Multilevel mediation analyses

**Figure 3.**
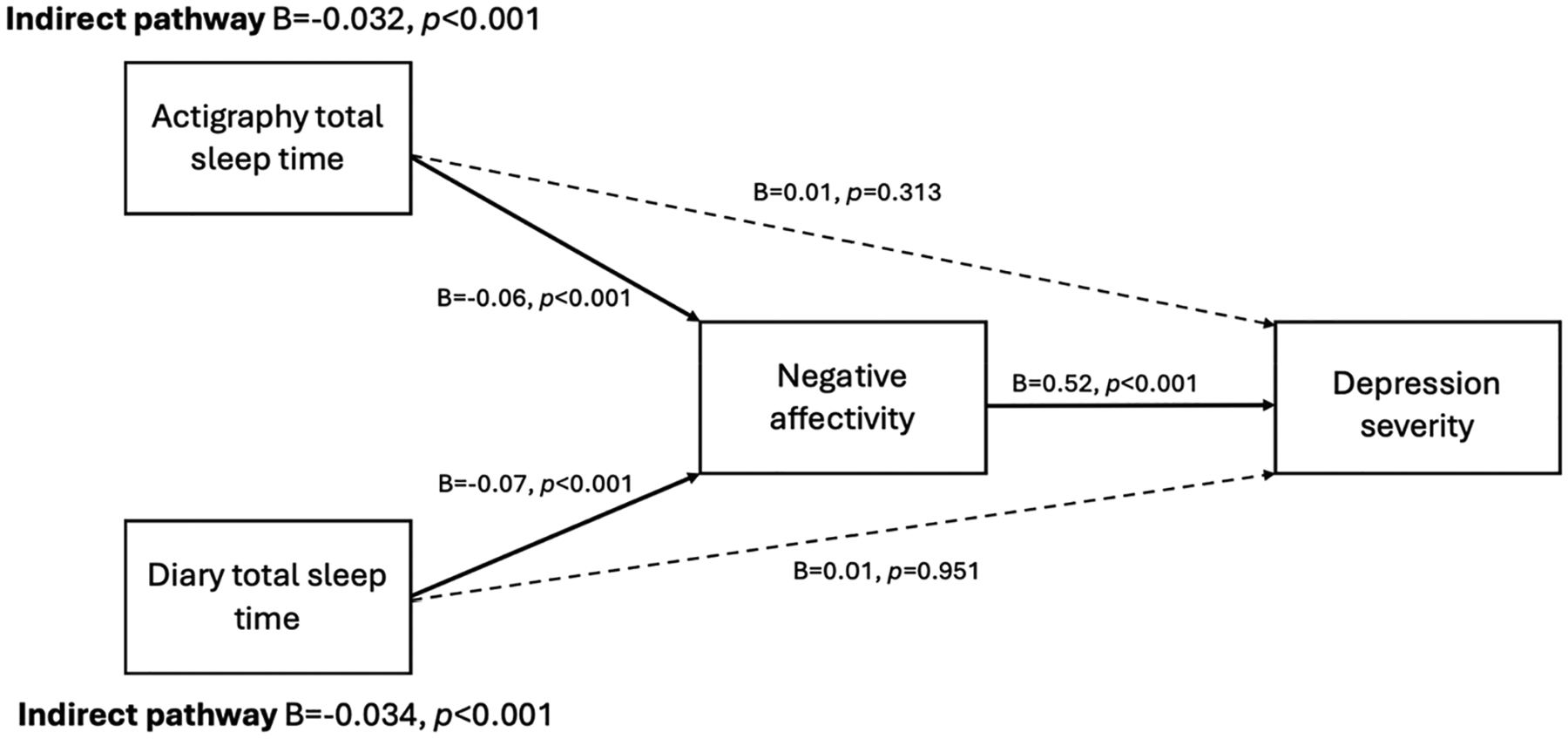
Multilevel mediation linking diary and actigraphy total sleep time with next day depression severity through negative affectivity to interpersonal events. Estimates represent standardized coefficients. Solid lines represent significant paths; dashed lines represent non-significant paths.

There was a significant within-person mediation for nightly changes in diary and actigraphy total sleep time impacting next-day depression through negative interpersonal affectivity (Figure 3). Sleeping less than usual was associated with greater next-day negative interpersonal affectivity, which in turn was associated with greater depression severity. There were no direct effects between within-person fluctuations in total sleep time and next-day depression after accounting for indirect effects through negative interpersonal affectivity. Removing negative (30%) and positive (18.6%) reported events that did not include family, peers, or other adults or removing young adults (N = 19) did not change any results.

## Discussion

The current study used an intensive longitudinal design to test changes throughout treatment and daily associations between sleep, depression severity, and interpersonal affectivity in adolescents and young adults with depression and suicidality engaged in intensive treatment. Over the course of treatment focused on depression and suicidality, daily self-reported and weekly clinician-reported depression improved, and self-reported sleep duration increased. Increased negative, but not positive, reactivity to interpersonal events mediated daily associations between sleep loss and increased depression. These findings controlled for prior depression and time in treatment, suggesting that within-person fluctuations in total sleep time impact next-day depression through increased negative affective reactivity above and beyond any improvements in sleep and depression throughout treatment. Of note, daily relationships between sleep and depression were amplified in participants with clinician-rated moderate-to-severe depression severity, suggesting greater sensitivity to sleep loss with greater overall depressive severity. Taken together, our findings suggest daily changes in total sleep time can contribute to depression, possibly through increased negative affective reactivity, and that the effects of sleep loss on mood may be most potent for those with clinically significant depression. Targeting sleep directly in higher acuity clinical samples may mitigate residual sleep symptoms and improve treatment outcomes.

Heightened negative affectivity to interpersonal events may be a proximal mechanism for how sleep loss impacts depression severity in a high-risk sample of clinically depressed adolescents and young adults. Our findings linking short sleep with increased negative affectivity are consistent with prior work in a subsample of the current study, which found that daily associations between short sleep and negative affectivity was associated with increased suicidal ideation [19]. Notably, the current study and the prior substudy both controlled for prior-day depression, suggesting that daily relationships between sleep loss and next-day negative affectivity is independent of prior depression. Negative affectivity may reflect impaired affective functioning, including altered mood states, heightened emotional responses, and poor emotion regulation [10]. Negative affectivity may be a shared underlying process linking disrupted sleep with both depression and suicidal ideation. While depression and suicidal ideation are interrelated and both impacted by disrupted sleep, there are also distinct pathways separating these processes [30], which may have not been captured in the present study design. Sleep loss can affect dys-regulation of corticolimbic circuits involved in affective processing [31]. For instance, sleep supports amygdala depotentiation for negative stimuli [32]. This process could be blunted by sleep loss and may be a potential pathway by which sleep loss impacts emotional interpretations of negative stimuli. These processes may be further impaired in adolescents and young adults with clinical depression [31], supporting our finding that the effects of sleep loss on next-day depression were most impairing for participants with moderate-to-severe clinical depression. Increased negative affectivity to interpersonal events following sleep loss may also be due to impaired ability to regulate emotion. In both a large nationally representative adolescent cohort and an adolescent in-patient sample, using fewer adaptive emotion regulation strategies (e.g., problem solving) and more maladaptive strategies (e.g., rumination and suppression) mediated associations between sleep problems and depression [33,34]. Given the intensive monitoring nature of the current study (M = 60 days daily monitoring), we limited the number of daily prompts to reduce participant burden. Future work examining nuances of affective functioning, including emotion regulation strategies and repeated time points across the day, may clarify the effects of sleep loss on affectivity more broadly and permit examination of potential diurnal effects.

Although we did not find evidence linking sleep loss with blunted positive affectivity to interpersonal situations, we may have been limited by a single daily time point. Laboratory and naturalistic studies in adults [35,36] support diurnal rhythmicity of PA, increasing in the morning, peaking in the afternoon, and decreasing during the evening. Blunted rhythmicity of PA may reflect anhedonia [37], and the rhythmicity of PA may be more responsive to sleep loss than a single time point [38]. In contrast, evidence supporting a diurnal rhythm in NA is mixed; NA may peak during the biological night but remains elevated throughout the day in depression [39]. In the current study, assessing retrospective NA and PA at one daily time point may have been sufficient to capture elevated NA following sleep loss but not blunted PA rhythmicity throughout the day.

While participants were engaged in intensive outpatient treatment focused on depression and suicidality, self-reported total sleep time increased throughout treatment. This is consistent with other work in adolescent depression showing improved subjective sleep after general psychotherapy treatment and at 1-year follow-up [22]. Despite sleep improvements, approximately half of that sample reported residual sleep problems at follow-up, and a third of participants with fully remitted depression reported continued sleep disturbances. Unfortunately, sleep disturbances, particular insomnia symptoms and short sleep, are one of the most common residual symptoms in adolescent depression [40]. In the current study, we did not see significant treatment changes in sleep duration measured with actigraphy. Sleep perception may be influenced by depressed mood states and not reflective of sleep-wake behaviors [41], possibly contributing to our modality specific findings in changes in sleep duration over time. The significant presence of residual sleep problems suggests current treatments for depression are not adequately targeting sleep. Sleep disturbances, particularly short sleep, are under-targeted mechanistic factors that may contribute to depression recurrence. Targeting sleep disturbances directly can reduce adolescent depression [42] and may be an underemphasized therapeutic component of intensive treatments. While the current study focused on sleep duration, sleep is multidimensional [43] and integrating behavioral treatments that address short and irregular sleep amount and timing, as well as insomnia symptoms, may be most impactful. The Transdiagnostic Sleep and Circadian intervention directly targets sleep and circadian disturbances and has been specifically designed for adolescents [44]. Preliminary work suggests improved regularity among depressed and suicidal youth [45]. Although currently limited to outpatient settings, adapting and implementing Transdiagnostic Sleep and Circadian intervention for higher acuity settings could test whether enhanced focus on sleep using evidence-based approaches directly improves clinical outcomes. Future studies should investigate whether adolescents with short sleep and insomnia symptoms benefit most from behavioral sleep treatments to improve depression [46].

Strengths of the current study include a large clinical sample of adolescents and young adults engaged in intensive treatment with ~7,700 days of analyzable data. Leveraging an intensive longitudinal design, we were able to examine within-person fluctuations in sleep across multiple months, providing naturalistic evidence for how sleep impacts depression on a day-to-day basis. Our analyses were limited by concurrent assessments of affective reactivity and depression symptoms during evening surveys, which limits temporal causality of our mediation analyses. The intensive nature of our monitoring period limited the scope of measuring daily affective functioning. Although study 1 and study 2 were similar in design, there were key differences in monitoring length (e.g., up to 2 or 3 months, respectively) and behavioral sleep assessment, which necessitated covarying for study in our analyses. While diverse in sexual orientation, our sample was predominantly White and female sex at birth, reflective of treatment seeking samples [47]. Youth with minoritized racial and gender identities may experience greater sleep difficulties and psychopathology than their peers [48,49] and may be missing from treatment samples.

## Supplementary Material

1

Supplementary data related to this article can be found at https://doi.org/10.1016/j.jadohealth.2026.02.009.

## Figures and Tables

**Table 1:** Baseline clinical and demographic information for the sample

Variables	Sample (N = 198)
Age, mean (SD) [range]	16.2 (2.0) [13–24]
Sex at birth, *n* (%)	
Female	151 (76%)
Male	47 (24%)
Race, *n* (%)	
Asian	10 (5%)
Black	13 (7%)
White	163 (82%)
Multiracial	12 (6%)
Ethnicity, *n* (%)	
Hispanic or Latino	12 (6%)
Sexual orientation	
Bisexual	54 (27%)
Gay or Lesbian	20 (10%)
Heterosexual	64 (33%)
Other orientation	42 (21%)
No information	18 (9%)
Secondary school	179 (90%)
Clinical presentation, *n* (%)	
Major depressive disorder	181 (91%)
Any anxiety disorder	149 (75%)
Generalized anxiety disorder	138 (70%)
Social anxiety	105 (53%)
Obsessive compulsive disorder	17 (9%)
Specific phobia	10 (5%)
Post-traumatic stress disorder	6 (3%)
Attention-Deficit/Hyperactive Disorder	66 (33%)
History of hypo/mania	26 (13%)
Insomnia	76 (38%)
Behavioral disorder	11 (5%)
Eating disorder	16 (8%)
Psychiatric medication	165 (83%)
Study variables, mean *(SD)*	
Daily depression	49.2 (23.9)
Negative interpersonal affectivity	54.4 (27.2)
Positive interpersonal affectivity	65.6 (23.8)
Diary total sleep time (hours)	7.9 (2.1)
Diary sleep onset	12:12 A.M. (2.1)
Actigraphy total sleep time (hours)	7.4 (1.9)
Actigraphy sleep onset	11:48 P. M. (2.8)

**Table 2:** Daily associations between changes in total sleep time and next-day depression and negative affectivity CI = 95% confidence intervals; SE = standard errors. Total sleep time was person-mean centered to reflect participant level averages (between TST) and daily fluctuations in total sleep time (within TST). Bolded values represent statistical significance.

Predictors	Daily depression								Negative interpersonal affectivity							
	Sleep diaries				Actigraphy				Sleep diaries				Actigraphy			
	Estimates	SE	CI	*p*	Estimates	SE	CI	*p*	Estimates	SE	CI	*p*	Estimates	SE	CI	*p*
Intercept	68.58	12.99	43.13–94.03	<.001	53.80	13.02	28.29–79.30	<.001	80.62	13.41	54.35–106.89	**<.001**	72.73	12.75	47.75–97.71	**<.001**
Age	−0.36	0.54	−1.42 to 0.70	.509	−0.31	0.55	−1.40 to 0.78	.580	−1.03	0.56	−2.13 to 0.06	.065	−0.83	0.55	−1.92 to 0.26	.135
Sex at birth	−5.07	2.41	−9.82 to −0.33	**.036**	−5.55	2.55	−10.58 to −0.52	**.031**	−6.50	2.46	−11.34 to −1.66	**.009**	−7.26	2.52	−12.22 to −2.31	**.004**
Time in study	−0.06	0.03	−0.11 to −0.00	**.044**	−0.06	0.03	−0.11 to 0.00	**.039**	0.08	0.02	0.04–0.13	**.001**	0.08	0.02	0.03–0.12	**.002**
Protocol	−1.11	2.27	−5.59 to 3.37	.625	−0.65	2.44	−5.46 to 4.16	.790	−1.81	2.31	−6.36 to 2.74	.434	−0.50	2.42	−5.26 to 4.26	.837
Weekend	−2.89	0.56	−3.99 to 1.80	**<.001**	−2.44	0.58	−3.59 to 1.30	**<.001**	−2.97	0.70	−4.34 to −1. 59	**<.001**	−3.14	0.73	−4.57 to −1. 71	**<.001**
Prior-day depression	0.10	0.01	0.08–0.13	**<.001**	0.11	0.01	0.08–0.13	**<.001**	0.10	0.02	0.07–0.14	**<.001**	0.13	0.02	0.10–0.17	**<.001**
Between TST	−1.91	0.94	−3.78 to −0.05	**.044**	−0.19	1.15	−2.46 to 2.09	.871	−1.72	0.98	−3.65 to 0.20	.079	−1.46	1.13	−3.69 to 0.76	.196
Within TST	−0.35	0.14	−0.62 to 0.08	**.010**	−0.23	0.16	−0.54 to 0.08	.149	−0.80	0.17	−1.13 to −0.46	**<.001**	−0.84	0.20	−1.23 to −0.46	**<.001**

Random effects				
Residu a1 (σ^2^)	341.79	347.51	514.35	515.82
Intercepl (τ_00_)	191.26	202.03	181.48	174.09
Slope (τ_11_)	0.06	0.05	0.02	0.02
Covariance (ρ_01_)	−0.33	−0.30	−0.34	−0.29
ICC	0.37	0.37	0.25	0.24
N	195	195	193	193
Observations	5594_days_	5189_days_	5305_days_	4899_days_

## Data Availability

Data are available upon reasonable request.
