# Supplementary material for: Sleeping Less Than Usual Is Associated With Greater Daily Depression and Higher Reactivity to Negative Interpersonal Events Among Suicidal Adolescents
Source: J Adolesc Health. Author manuscript; Available in PMC 2026 May 27. (PMC13215565; doi:10.1016/j.jadohealth.2026.02.009)

**Supplemental Table 1.** Changes in daily self-reported depression and weekly clinician-rated depression throughout time enrolled in treatment.

|  | **Daily Diary Depression** | | | | **Weekly Clinician-Rated Depression** | | | |  |
| --- | --- | --- | --- | --- | --- | --- | --- | --- | --- |
| *Predictors* | *Estimates* | *SE* | *CI* | *p* | *Estimates* | *SE* | *CI* | *p* |  |
| Intercept | 54.94 | 10.08 | 35.20 – 74.69 | <0.001 | 3.28 | 0.65 | 2.00 – 4.56 | <0.001 |  |
| Age | -0.17 | 0.58 | -1.31 – 0.98 | 0.775 | 0.07 | 0.04 | -0.01 – 0.14 | 0.068 |  |
| Sex at birth | -3.98 | 2.63 | -9.16 – 1.21 | 0.132 | -0.19 | 0.17 | -0.52 – 0.15 | 0.270 |  |
| Time in study | -0.06 | 0.03 | -0.12 – -0.01 | **0.025** | -0.09 | 0.01 | -0.11 – -0.07 | **<0.001** |  |
| Protocol | -0.33 | 2.51 | -5.29 – 4.63 | 0.896 | -0.22 | 0.16 | -0.54 – 0.10 | 0.174 |  |
| Weekend | | -2.58 | 0.49 | -3.53 – -1.63 | **<0.001** |  |  |  |  |
| **Random Effects** | | | | | | | | |  |
| Residual (σ^2^) | 346.44 | | | | 0.28 | | | |  |
| Intercept (τ_00_) | 241.15 | | | | 0.89 | | | |  |
| Slope (τ_11_) | 0.08 | | | | 0.01 | | | |  |
| Covariance (ρ_01_) | -0.25 | | | | 0.04 | | | |  |
| ICC | 0.44 | | | | 0.83 | | | |  |
| N | 198 | | | | 195 | | | |  |
| Observations | 7339_days_ | | | | 1421_weeks_ | | | |  |

**Supplemental Table 2.** Changes in diary and actigraphy total sleep time throughout time enrolled in treatment.

|  | **Diary Total Sleep Time** | | | | **Actigraphy Total Sleep Time** | | | |
| --- | --- | --- | --- | --- | --- | --- | --- | --- |
| *Predictors* | *Estimates* | *SE* | *CI* | *p* | *Estimates* | *SE* | *CI* | *p* |
| Intercept | 9.34 | 0.66 | 8.04 – 10.65 | **<0.001** | 7.47 | 0.59 | 6.32 – 8.62 | **<0.001** |
| Age | -0.11 | 0.04 | -0.19 – -0.04 | **0.004** | -0.04 | 0.03 | -0.10 – 0.03 | 0.261 |
| Sex at birth | -0.16 | 0.17 | -0.50 – 0.18 | 0.359 | -0.47 | 0.15 | -0.77 – -0.17 | **0.003** |
| Time in study | 0.01 | 0.00 | 0.00 – 0.01 | **<0.001** | 0.00 | 0.00 | -0.00 – 0.00 | 0.565 |
| Protocol | -0.13 | 0.16 | -0.45 – 0.19 | 0.426 | 0.62 | 0.15 | 0.33 – 0.90 | **<0.001** |
| Weekend | 0.88 | 0.05 | 0.79 – 0.98 | **<0.001** | 0.71 | 0.04 | 0.62 – 0.79 | **<0.001** |
| **Random Effects** | | | | | | | | |
| Residual (σ^2^) | 3.45 | | | | 2.85 | | | |
| Intercept (τ_00_) | 1.15 | | | | 0.74 | | | |
| Slope (τ_11_) | 0.00 | | | | 0.00 | | | |
| Covariance (ρ_01_) | -0.60 | | | | -0.20 | | | |
| ICC | 0.21 | | | | 0.21 | | | |
| N | 197 | | | | 198 | | | |
| Observations | 7276_days_ | | | | 7671_days_ | | | |

**Supplemental Table 3.** Changes in diary and actigraphy sleep onset throughout time enrolled in treatment.

|  | **Diary Sleep Onset** | | | | **Actigraphy Sleep Onset** | | | |
| --- | --- | --- | --- | --- | --- | --- | --- | --- |
| *Predictors* | *Estimates* | *SE* | *CI* | *p* | *Estimates* | *SE* | *CI* | *p* |
| Intercept | 19.94 | 0.92 | 18.13 – 21.74 | **<0.001** | 20.41 | 0.84 | 18.77 – 22.05 | **<0.001** |
| Age | 0.24 | 0.05 | 0.13 – 0.34 | **<0.001** | 0.20 | 0.05 | 0.10 – 0.29 | **<0.001** |
| Sex at birth | 0.53 | 0.24 | 0.06 – 1.00 | **0.029** | 0.43 | 0.22 | -0.00 – 0.85 | 0.051 |
| Time in study | -0.00 | 0.00 | -0.00 – 0.00 | 0.799 | -0.00 | 0.00 | -0.01 – 0.01 | 0.923 |
| Protocol | 0.30 | 0.23 | -0.15 – 0.75 | 0.189 | 0.03 | 0.21 | -0.38 – 0.44 | 0.889 |
| Weekend | 0.61 | 0.04 | 0.53 – 0.69 | **<0.001** | 0.60 | 0.06 | 0.49 – 0.72 | **<0.001** |
| **Random Effects** | | | | | | | | |
| Residuals (σ^2^) | 2.23 | | | | 5.64 | | | |
| Intercept (τ_00_) | 2.21 | | | | 1.93 | | | |
| Slope (τ_11_) | 0.00 | | | | 0.00 | | | |
| Covariance (ρ_01_) | -0.39 | | | | -0.52 | | | |
| ICC | 0.48 | | | | 0.25 | | | |
| N | 197 | | | | 198 | | | |
| Observations | 7277_days_ | | | | 7671_days_ | | | |

**Supplemental Table 4.** Changes in diary and actigraphy wake time throughout time enrolled in treatment.

|  | **Diary Waketime** | | | | **Actigraphy Waketime** | | | |
| --- | --- | --- | --- | --- | --- | --- | --- | --- |
| *Predictors* | *Estimates* | *SE* | *CI* | *p* | *Estimates* | *SE* | *CI* | *p* |
| Intercept | 5.20 | 0.82 | 3.59 – 6.81 | **<0.001** | 4.61 | 0.89 | 2.86 – 6.35 | **<0.001** |
| Age | 0.15 | 0.05 | 0.05 – 0.24 | **0.002** | 0.20 | 0.05 | 0.10 – 0.30 | **<0.001** |
| Sex at birth | 0.30 | 0.21 | -0.12 – 0.72 | 0.163 | 0.25 | 0.23 | -0.20 – 0.71 | 0.273 |
| Time in study | 0.01 | 0.00 | 0.00 – 0.01 | **0.025** | 0.01 | 0.00 | 0.00 – 0.01 | **0.007** |
| Protocol | 0.16 | 0.21 | -0.25 – 0.56 | 0.439 | 0.30 | 0.22 | -0.14 – 0.74 | 0.177 |
| Weekend | 1.48 | 0.04 | 1.40 – 1.57 | **<0.001** | 1.48 | 0.05 | 1.39 – 1.57 | **<0.001** |
| **Random Effects** | | | | | | | | |
| Residuals (σ^2^) | 2.64 | | | | 3.35 | | | |
| Intercept (τ_00_) | 1.54 | | | | 1.94 | | | |
| Slope (τ_11_) | 0.00 | | | | 0.00 | | | |
| Covariance (ρ_01_) | -0.20 | | | | -0.32 | | | |
| ICC | 0.40 | | | | 0.39 | | | |
| N | 197 | | | | 198 | | | |
| Observations | 7276_days_ | | | | 7671_days_ | | | |

Note: Delaying of wake times throughout treatment persisted after accounting for summer vs. non-summer changes in sleep schedules from school.

**Supplemental Table 5.** Daily associations between sleep onset and self-reported depression

|  | **Diary Sleep Onset** | | | | **Actigraphy Sleep Onset** | | | |
| --- | --- | --- | --- | --- | --- | --- | --- | --- |
| *Predictors* | *Estimates* | *SE* | *CI* | *p* | *Estimates* | *SE* | *CI* | *p* |
| Intercept | 34.98 | 17.06 | 1.55 – 68.40 | **0.040** | 57.18 | 19.52 | 18.95 – 95.42 | **0.003** |
| Age | -0.38 | 0.56 | -1.47 – 0.72 | 0.497 | -0.26 | 0.57 | -1.38 – 0.87 | 0.650 |
| Sex at birth | -5.42 | 2.45 | -10.26 – -0.58 | **0.028** | -5.36 | 2.53 | -10.35 – -0.38 | **0.035** |
| Time in study | -0.06 | 0.03 | -0.11 – -0.00 | **0.037** | -0.06 | 0.03 | -0.11 – -0.00 | **0.039** |
| Weekend | -3.24 | 0.56 | -4.33 – -2.15 | **<0.001** | -2.60 | 0.58 | -3.73 – -1.47 | **<0.001** |
| Protocol | -0.94 | 2.29 | -5.45 – 3.57 | 0.682 | -0.77 | 2.34 | -5.38 – 3.84 | 0.743 |
| Prior-day Depression | 0.10 | 0.01 | 0.08 – 0.13 | **<0.001** | 0.11 | 0.01 | 0.08 – 0.13 | **<0.001** |
| Between sleep onset | 0.79 | 0.71 | -0.60 – 2.19 | 0.264 | -0.23 | 0.81 | -1.83 – 1.38 | 0.780 |
| Within sleep onset | 0.15 | 0.17 | -0.18 – 0.49 | 0.372 | 0.00 | 0.11 | -0.22 – 0.22 | 0.994 |
| **Random Effects** | | | | | | | | |
| Residuals (σ^2^) | 341.77 | | | | 347.61 | | | |
| Intercept (τ_00)_ | 198.14 | | | | 202.34 | | | |
| Slope (τ_11_) | 0.06 | | | | 0.05 | | | |
| Covariance (ρ_01_) | -0.35 | | | | -0.30 | | | |
| ICC | 0.37 | | | | 0.37 | | | |
| N | 196 | | | | 195 | | | |
| Observations | 5604_days_ | | | | 5189_days_ | | | |

**Supplemental Table 6.** Daily associations between total sleep time and positive affectivity to interpersonal events.

|  | **Diary Total Sleep Time** | | | | **Actigraphy Total Sleep Time** | | | |
| --- | --- | --- | --- | --- | --- | --- | --- | --- |
| *Predictors* | *Estimates* | *SE* | *CI* | *p* | *Estimates* | *SE* | *CI* | *p* |
| Intercept | 80.67 | 13.56 | 54.11 – 107.23 | **<0.001** | 89.88 | 13.24 | 63.95 – 115.81 | **<0.001** |
| Age | -1.17 | 0.56 | -2.28 – -0.07 | **0.038** | -1.39 | 0.56 | -2.50 – -0.28 | **0.014** |
| Sex at birth | 2.71 | 2.51 | -2.25 – 7.66 | 0.283 | 3.41 | 2.60 | -1.70 – 8.53 | 0.190 |
| Time in study | 0.02 | 0.03 | -0.04 – 0.09 | 0.445 | 0.01 | 0.03 | -0.06 – 0.07 | 0.837 |
| Weekend | 4.23 | 0.58 | 3.10 – 5.36 | **<0.001** | 4.51 | 0.61 | 3.33 – 5.70 | **<0.001** |
| Protocol | -0.87 | 2.38 | -5.56 – 3.82 | 0.715 | -1.18 | 2.49 | -6.09 – 3.73 | 0.636 |
| Prior-day Depression | -0.04 | 0.01 | -0.07 – -0.01 | **0.004** | -0.04 | 0.01 | -0.07 – -0.02 | **0.002** |
| TST between | 0.54 | 0.98 | -1.39 – 2.47 | 0.582 | -0.18 | 1.17 | -2.48 – 2.13 | 0.879 |
| TST within | -0.05 | 0.14 | -0.33 – 0.22 | 0.709 | -0.24 | 0.17 | -0.57 – 0.08 | 0.140 |
| **Random Effects** | | | | | | | | |
| Residuals (σ^2^) | 355.15 | | | | 366.97 | | | |
| Intercept (τ_00_) | 199.85 | | | | 195.55 | | | |
| Slope (τ_11_) | 0.08 | | | | 0.09 | | | |
| Covariance (ρ_01_) | -0.27 | | | | -0.23 | | | |
| ICC | 0.40 | | | | 0.40 | | | |
| N | 195 _ID_ | | | | 195 _ID_ | | | |
| Observations | 5472_days_ | | | | 5071_days_ | | | |

|  | **Study 1: Actigraphic TST** | | | | **Study 2: Actigraphic TST** | | | |
| --- | --- | --- | --- | --- | --- | --- | --- | --- |
| *Predictors* | *B* | *SE* | *CI* | *p* | *B* | *SE* | *CI* | *p* |
| (Intercept) | 7.49 | 0.73 | 6.05 – 8.92 | **<0.001** | 8.15 | 0.83 | 6.53 – 9.77 | **<0.001** |
| Age | -0.04 | 0.04 | -0.13 – 0.04 | 0.310 | -0.04 | 0.05 | -0.14 – 0.06 | 0.448 |
| Sex [Male] | -0.07 | 0.26 | -0.59 – 0.45 | 0.788 | -0.63 | 0.18 | -1.00 – -0.27 | **0.001** |
| Weekend | 0.61 | 0.07 | 0.48 – 0.74 | **<0.001** | 0.76 | 0.06 | 0.65 – 0.87 | **<0.001** |
| Day Number | 0.00 | 0.00 | -0.00 – 0.00 | 0.772 | 0.00 | 0.00 | -0.00 – 0.00 | 0.591 |
| Summer | 0.04 | 0.13 | -0.22 – 0.29 | 0.779 | -0.08 | 0.11 | -0.29 – 0.14 | 0.478 |
| **Random Effects** | | | | | | | | |
| σ^2^ | 2.37 | | | | 3.11 | | | |
| τ_00_ | 0.63 _ID_ | | | | 0.79 _ID_ | | | |
| τ_11_ | 0.00 _ID.DayNumber_ | | | | 0.00 _ID.DayNumber_ | | | |
| ρ_01_ | 0.01 _ID_ | | | | -0.36 _ID_ | | | |
| ICC | 0.24 | | | | 0.19 | | | |
| N | 59 | | | | 139 | | | |
| Observations | 2726 | | | | 4945 | | | |

**Supplemental Table 7.** Study separate comparisons of actigraphic total sleep time

**Figure S1.** Histograms of VAS scales from evening surveys.


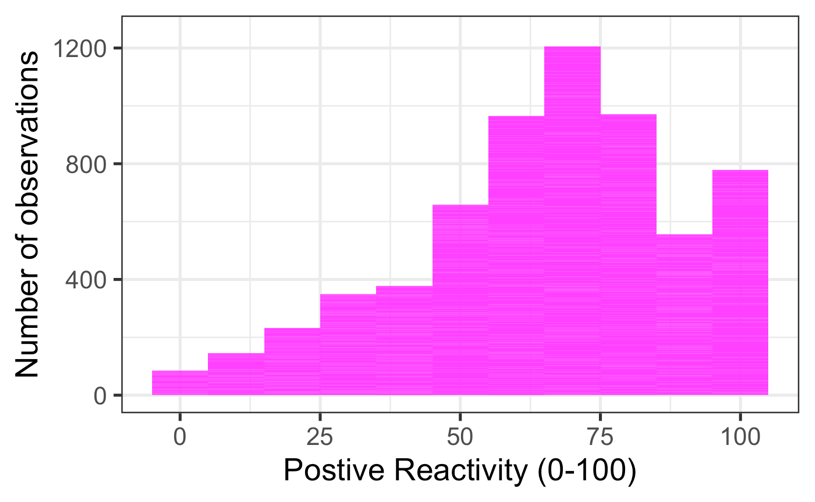

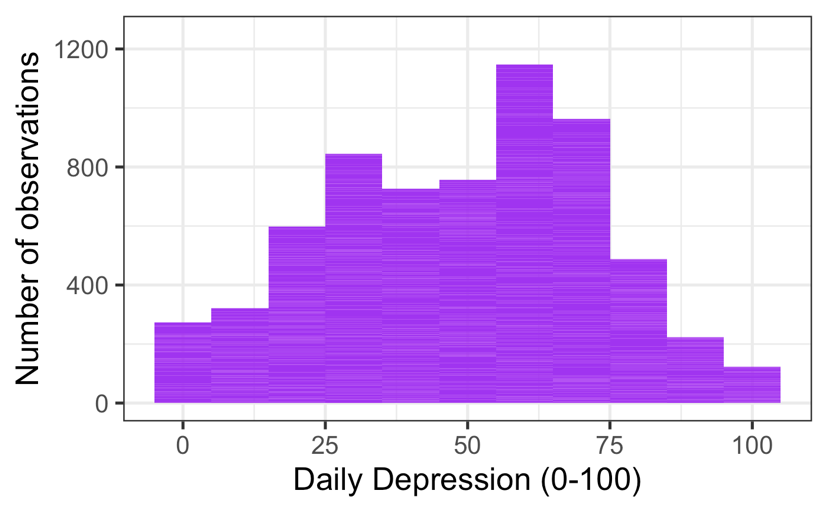

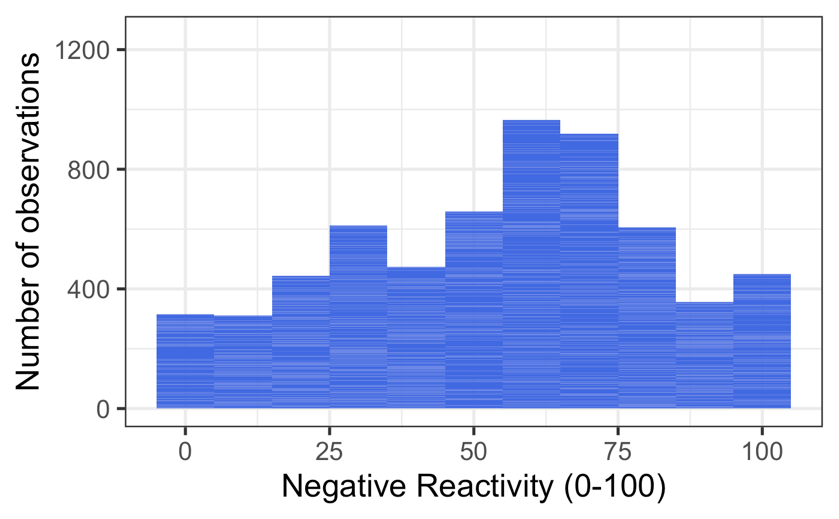

Supplement: 1 [file NIHMS2178335-supplement-1.docx]
